# Effects of variability of practice in music: a pilot study on fast goal-directed movements in pianists

**DOI:** 10.3389/fnhum.2014.00598

**Published:** 2014-08-11

**Authors:** Marc Bangert, Anna Wiedemann, Hans-Christian Jabusch

**Affiliations:** Institute of Musicians’ Medicine (IMM), University of Music Carl Maria von Weber DresdenDresden, Germany

**Keywords:** variability of practice, piano, motor skill acquisition, musicians, schema theory

## Abstract

Variability of Practice (VOP) refers to the acquisition of a particular target movement by practicing a range of varying targets rather than by focusing on fixed repetitions of the target only. VOP has been demonstrated to have beneficial effects on transfer to a novel task and on skill consolidation. This study extends the line of research to musical practice. In a task resembling a barrier-knockdown paradigm, 36 music students trained to perform a wide left-hand interval leap on the piano. Performance at the target distance was tested before and after a 30-min standardized training session. The high-variability group (VAR) practiced four different intervals including the target. Another group (FIX) practiced the target interval only. A third group (SPA) performed spaced practice on the target only, interweaving with periods of not playing. Transfer was tested by introducing an interval novel to either group. After a 24-h period with no further exposure to the instrument, performance was retested. All groups performed at comparable error levels before training, after training, and after the retention (RET) interval. At transfer, however, the FIX group, unlike the other groups, committed significantly more errors than in the target task. After the RET period, the effect was washed out for the FIX group but then was present for VAR. Thus, the results provide only partial support for the VOP hypothesis for the given setting. Additional exploratory observations suggest tentative benefits of VOP regarding execution speed, loudness, and performance confidence. We derive specific hypotheses and specific recommendations regarding sample selection and intervention duration for future investigations. Furthermore, the proposed leap task measurement is shown to be (a) robust enough to serve as a standard framework for studies in the music domain, yet (b) versatile enough to allow for a wide range of designs not previously investigated for music on a standardized basis.

## Introduction

Playing music is a complex motor skill orchestrated by sequences of goal-directed movements. Given the overall difficulty of the skill and the volume of the pool of motor programs to be mastered, the efficiency of practice in the allocated practice time has to be optimized. In this respect, findings from motor learning research may be applicable to facilitate learning of musical instruments. Several concepts and parameters regarding the design of learning schedules have been introduced to research, specifically variability (Schmidt, 1975) and distribution of practice (Adams and Reynolds, 1954).

Schmidt’s (1975) “schema theory” formulates a *schema* as an abstract code for a class of movements with a common pattern. Schema learning, then, is the gradual formation of a central prototype from a number of specific experiences within a motor class. Introducing variability in practice therefore is a key concept as it relates to the idea of generalization of movement patterns. Regarding the potential benefits of variability, two contrasting hypotheses have been proposed:

- The specificity of practice hypothesis (SOP; Henry, 1968; Tulving and Thompson, 1973) states that the conditions during practice should match the conditions during retrieval/performance as closely as possible. From this view, introducing variability into the practice schedule would only be recommended if the respective skill requires the ability to generate highly adaptive and flexible outcomes. In terms of making music, this would be the case e.g., for jazz improvisation. Conversely, a skill like playing classical poses high demands on exact reproduction of motor sequences for successful performance. In the latter, the SOP hypothesis predicts learning facilitation in a practice schedule matched to the performance demand, i.e., a fixed practice schedule.
- Schmidt (1975) schema theory implicates the contrasting variability of practice hypothesis (VOP), which holds that practicing with task variations is beneficial not only for flexible but also for fixed performance scenarios. According to the theory, the latter benefits would be realized through the formation of a stronger schema between motor parameters and performance outcome, reflected in improved learning and facilitated generalization to novel parameter sets. This idea would contrast with approaches in which the student would be encouraged to focus on mastering a specific target motor program within narrow outcome tolerance before moving on to new tasks.

Empirical evidence for the validity of the VOP hypothesis has been provided by a considerable number of studies. Although originally reported for verbal tasks (Battig, 1966, 1972), research has since been extended to motor skill learning, which today is a primary focus of VOP studies (for reviews, see Shapiro and Schmidt, 1982; Van Rossum, 1990; Wulf and Schmidt, 1997).

The VOP predictions as well as the supporting empirical effects seem counter-intuitive to previous “common-sense” ideas about effective practice scheduling. Yet the impact of the implications of variability approaches for teaching and instruction of practice structures in real-world settings has soon been recognized. Accordingly, research along the lines of variability schedules has been taken out of laboratory settings early on, and is now established for a broad spectrum of ecologically valid paradigms, ranging from physical education (e.g., Saemi et al., 2012) and sports (e.g., Douvis, 2005; Travlos, 2010) to clinical rehabilitation (e.g., Dick et al., 2000; Panarese et al., 2012).

Surprisingly, only few empirical accounts so far have investigated whether or not the effects are also applicable to motor practice of musical instruments or the musical voice. A number of studies (Welch, 1985; Rose, 2006; Stambaugh and Demorest, 2010; Stambaugh, 2011) are noteworthy in this respect. Welch (1985) conducted a study with 66 children between 7 and 9 years of age who listened to a target pitch and subsequently had to try to sing the note in tune, matching the pitch previously heard. The training session consisted of practicing the target pitch only (low variability group) or variably practicing six different pitches not including the target (high variability group). Each group was divided into three subgroups receiving (a) real-time visual feedback on their own vocal pitch via an oscilloscope including the target pitch indicated as reference line; (b) visual feedback like (a) but without a reference; or (c) no visual feedback at all. Welch (1985) found significant overall effects for the factor feedback but not for variability. Only when the no-feedback group (c) was analyzed separately, an effect of variability was emergent: the variable group attained significantly higher accuracy than the fixed group. This reflects superior learning in the variable group but is not fully consistent with the VOP hypothesis.

With respect to instrumental practice, Stambaugh and Demorest (2010) conducted experiments with 19 seventh-grade woodwind students who received a single 18-min session of rehearsing three unfamiliar songs under different instructional setting. One group practiced each song in separate blocks of 6 min each (low variability), the second group switched songs every 2 min (mid variability), the third group every minute (high variability). Performance measurements were taken immediately after practice (“acquisition”) and 1 day later (“RET”). With respect to technical accuracy, Stambaugh found no significant main effects or interactions. In a similar study, Stambaugh (2011) found the variable group to perform significantly faster than the blocked group. In both studies, due to the nature of the task, no pre-training assessment of actual task performance was possible. Thus, statistical comparisons were only possible between-groups after the fact.

Consequently, the present study aims at studying a motor skill less complex than melodic sequences, which allows for an assessment of technical accuracy preceding the practice intervention. We chose a time-constrained wide downward interval leap with the left hand on the piano. The task *per se* resembles a simple goal-directed reaching movement, making immediate measures of accuracy (like distance errors) feasible even in subjects with no previous exposure to the specific task. On the other hand, proper execution is challenging enough to leave ample space for improvement through practice, i.e., ceiling effects were unlikely. The same reasoning led to choosing (a) the left hand as the motor executor (since in classical Western piano literature, the overall training is reduced for the left hand compared to the right hand, Kopiez et al., 2012); and (b) participants who study piano as their minor subject. Furthermore, the wide interval leap is an ideal task as it is experimentally simple enough to allow for direct comparisons with previous research in motor learning outside the musical field, while at the same time being close to musical reality: the task bears ecological validity as it appears in the classical piano (e.g., the opening bars of Ludwig van Beethoven’s Piano Sonata c-minor, op. 111) and jazz piano (e.g., stride piano styles) repertoire. Yet, it appears not too frequently hence it does not belong to the category of highly overlearned motor patterns in piano playing.

The empirical approach in this study exploits a classic experiment conducted by McCracken and Stelmach (1977) as a template for an ecologically valid experimental setup with musicians. McCracken and Stelmach (1977) presented a task involving time-constrained hand movements to targets at defined distances. The training phase consisted of 300 trials on four randomly alternating distances for one group, and of 300 trials on just one distance for another group. Our aim was to utilize a similar leap motion (musical interval on a piano as outlined above) in order to test whether or not the predictions made from VOP can be extended to musical practice in the given setting. Therefore, three groups of varying degrees of variability and intensity of practice were established.

Adopted to the piano paradigm, the VOP hypothesis leads to the following predictions: higher VOP is associated with (1) similar or higher error rates after acquisition but (2a) lower error rates at RET and (2b) at transfer of the skill to a novel target.

In addition to the primary hypotheses as derived from error rate measurements in previous motor learning research, our secondary aim is to exploit the dataset as observational pilot material in order to derive hypotheses for further variables that are closely tied to musical precision and expressiveness. These hypotheses may serve as a starting point for future empirical approaches in this young area of motor learning research.

## Methods

### Participants

Thirty-six right-handed music students took part in the experiment (16 female; age 20.5 ± 2.2 years). All participants studied piano as their minor subject. Demographic information, handedness (Oldfield, 1971), practice habits (e.g., frequency of practice, percentage of technical exercises, preferred piano literature, etc.), and musical biography (e.g., age of commencement of piano and primary instrument, weekly hours of practice during different phases of childhood and adolescence) were obtained through a questionnaire. Left hand span was measured as the active span from thumb to little finger. Participants were randomly assigned to one of three experimental groups. Based on parameters related to expertise and physiological conditions (see Table 1), no differences were seen between groups (Mann-Whitney, all *p*-values > 0.05). Prior to the experiment, written informed consent was obtained from all participants. The experiments were performed in accordance with relevant institutional and national regulatory standards.

**Table 1:** **Overall demographic information of the participant sample**.

	**Median**	**Lower Quartile**	**Upper Quartile**
Age at time of experiment (years)	19.7	18.8	21.5
Age at commencement of piano practice (years)	13.0	9.3	15.8
Cumulative years of piano practice	6.1	3.6	10.0
Cumulative hours of piano practice	824	490	1413
Handedness (LQ)	100	98.5	100
Active digit 1–5 span (left hand) (mm)	197	190	217

*LQ designates the Laterality Quotient as defined by Oldfield (1971), a scale ranging from −100 (full left-handedness) to +100 (full right-handedness)*.

### Materials

**Figure 1:**
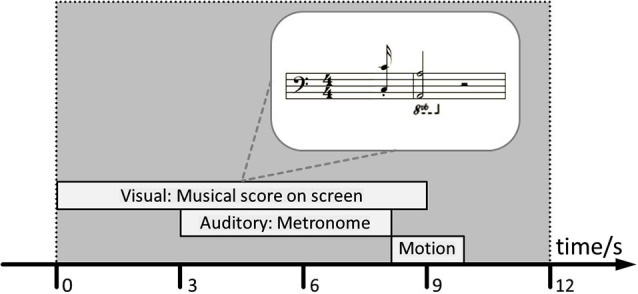
**Schematic representation of the time course of one single 12-s-trial**. The identical trial layout was used in the performance assessment and during the training session, respectively. The inset in the upper right corner depicts an example of the visual presentation of each task on the screen in the experimental setup.

The experiment was performed on a digital piano (Kawai MP8 II, KAWAI Musical Instruments Mfg. Co., Ltd., Japan). Standardized instructions and tasks were presented on a monitor placed in front of the subjects using Presentation® software (Version 16.1).^1^ Musical tasks were presented as musical notation (bass clef, one 4/4 measure with a lead-in upbeat; cf. Figure 1). Performance data were collected via the Kawai built-in MIDI-USB interface into MIDI files using custom-made recording software, and into Presentation® log files. Participants completed the computer-interactive procedure without an experimenter in the room. In order to ensure compliance with the instructions, the entire procedure was videotaped.

### Procedure

The core task was to train to perform a wide interval leap on the piano with the left hand.

The first musical event (upbeat), at the starting position of the leap, consisted of two notes (with an octave distance between them) to be played simultaneously by the thumb and little finger, respectively (see notation in Figure 1). Similarly, the second event (downbeat), at the leap destination, consisted of another two notes (again with an octave distance between them). Thus, the leap movement had to be executed by relocating the entire wrist and hand, so that differences in fingering as a confound could be excluded.

All intervals used as target, transfer, and training tasks were chosen from the diatonic C-Major scale so that all leaps were to be played on white keys only. In the time domain, the training goal was to execute the metronome-guided leap in 187.5 ms—a semiquaver at a tempo of 80 beats per minute (BPM). The metronome provided a two-measure count-in but did not continue to provide an auditory pace once the movement was initiated.

#### Assessment of performance

**Figure 2:**
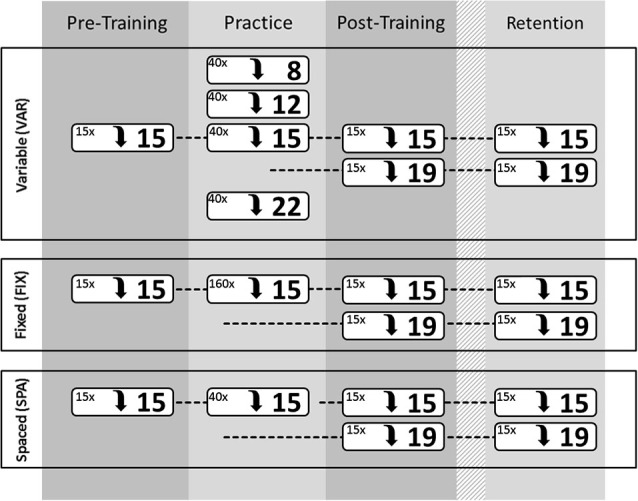
**Schematic summary of the test and training paradigm for the three groups VAR (top), FIX (middle), and SPA (bottom)**. The Pre-Training (PRE), post-Training (POST), and Retention (RET) tests were identical for each group. However, the actual training paradigm differed between the groups (see text). Each of the white boxes indicates the interval to be performed (distance in semitones; bold numbers on the right of each box) and the number of individual trials (top left of each box) with this interval.

The three experimental groups (Figure 2) performed identical pre-training (PRE), post-training (POST, immediately after training), and retention (RET, 24 h after training) tests, consisting of: (a) 15 metronome-guided repetitions of a target leap (TGT, spanning 15 semitones, a musical tenth); and (b) 15 repetitions on a transfer task in the POST and RET tests only, introducing an interval novel to either group (TSF, spanning 19 semitones). Participants were instructed to attempt to not only play the correct keys, but also accomplish correct timing (as specified by rhythmic notation and metronome tempo) and homogenous keystroke loudness (i.e., to avoid that the downbeat of the leap would be noticeably louder, or softer, respectively, compared to the upbeat). Before each performance assessment, a 2-min warm-up on the piano was granted. The choice of warm-up drills was free to each participant but excluded leap movements.

#### Training paradigm

Between PRE and POST testing, participants underwent a 30-min standardized computer-interactive training session (160-trials; each trial = one metronome-guided interval leap). Each of the three groups received a different practice schedule (Figure 2):

**FIX group:** Participants in the **fixed** group underwent a massed practice of the target interval only, thereby repeating it 160 times.
**VAR group:** These participants received **variable** training on the diatonic intervals 8, 12, 15, and 22 semitones, respectively, thereby spending 25% of their trials on each interval. The 40 instances of each interval were administered in a blocked-random order: intervals were presented in small blocks of five identical intervals each; the block order was randomized; yet immediate reruns of identical blocks were avoided. While the actual target interval was part of the item pool, the transfer interval was omitted.
**SPA group:** Participants in the **“spaced”** group practiced the target interval only, but (unlike the FIX group) only for a total of 40 trials. The trials were bundled to blocks of five each (like the VAR group). These 1-min blocks were sparsely presented with intermittent 3-min phases of not playing. During these 3-min resting phases, the participants were instructed to read portions of a short story. Focus of attention on the reading was established by informing the subject to be quizzed on details of the content later (the quiz consisting of three simple multiple-choice questions).

After each block, a screen display prompted the subjects to start the subsequent block actively by pressing the left piano foot pedal, providing the opportunity to insert moments of rest when needed. Besides the actual training intervention, participants gave written consent that on the day of the PRE measurement and during the period between POST and RET assessment (approximately 24 h), no further practice or other exposure to keyboard instruments was allowed.

### Analysis and statistics

2700 trials from PRE, POST, and RET assessments were recorded. Of these, 2549 were included in the analysis after discarding unanalyzable trials (e.g., trials during which the motion was not executed at all within the allotted trial time did not allow for the calculation of secondary variables). Data from the training phases were not included. Statistical tests were done using SPSS 19 (IBM).

The MIDI and Presentation® logfile data were consolidated into single ASCII raw data files. These were subsequently preprocessed in order to extract first level (within-subject) sums (primary variable) or medians (secondary variables), respectively, for each phase (PRE, POST, RET), condition (TGT, TSF), and subject.

#### Primary variable (error rate)

The *primary variable* of interest to assess musical performance precision (as formulated in the a priori hypotheses) was the overall number of missed and incorrectly played notes at the time points PRE, POST, and RET, hence indirectly reflecting spatial accuracy of the leap movement. The Median Error Score (MES) aimed at quantifying all relevant aspects of hitting a prescribed note correctly (or failing to do so) within the allocated time window. It was defined as the total count of all errors across all trials of each condition for each participant. The following events added to the gross error score, equally weighted: any required note that was missed; any slip note (additional notes in immediate spatiotemporal vicinity to otherwise correctly played notes); any additional wrong note within a time window from 500 ms before nominal onset to 500 ms after nominal end of the leap movement.

Statistical comparisons for specific hypotheses on group level were carried out using Mann-Whitney tests (between groups) and Wilcoxon tests (between sessions and between TGT/TSF within groups). Reported tests were Bonferroni-corrected for familywise Type I errors due to multiple comparisons. Global alpha was set at 0.05 level.

Timing is a possible confound for error rates, because e.g., the more slowly a subject would play (i.e., the more time one would grant oneself to execute the movement) the better the chances of avoiding wrong notes. Therefore, it was important to objectify that no systematic timing differences would account for any of the apparent error differences (LET, see below).

#### Secondary variables

This study is the first of its kind adopting the described motor learning paradigm to a musical context. Therefore, a series of possibly relevant *secondary variables* were defined, derived from the MIDI data, and subjected to an exploratory data analysis encompassing visual inspection of data. Based on these considerations, the following secondary metrics will be reported as promising candidates for hypothesis-generations and inferential tests in future research:

- **Leap Execution Time (LET)**. LET is calculated (in milliseconds) between the times of the start point and end of the leap movement, i.e., the time needed to travel the interval distance. Since at each position two notes were played in octave grip almost simultaneously, the mid-time point of the two note onsets was considered, respectively. The LET metric is of relevance since the metronome-guided time goal for the leap was 187.5 ms (1/16 note at 80 BPM). This ambitious goal was deliberately set to be not within reach for any one of the subjects (showing average times of ~280 ms). Hence, achieving faster leap times while preserving low error scores serves an additional indicator of training success.
- **Leap Loudness (LL)**: the MIDI key velocities of the end position of the leap. LL indicates how loud the musical downbeat was being played; analogue to the LET, the key velocities of both notes of the octave grip were averaged.
- **Leap Loudness Homogeneity (LLH)**: the average difference between the MIDI key velocities of the end position of the leap (musical downbeat) and the starting position of the leap (upbeat), respectively; as for the LET, both notes of the octave grip were averaged. LLH is different from LL as it independently quantifies the degree of loudness homogeneity across the movement (regardless of whether the leap was being played loudly or softly).

Due to the exploratory selection of variables, and due to the pioneering nature of the data (no previous research is available upon which to build hypotheses for the specific musical leap task), the present work will report these secondary data on a descriptive statistical level only.

## Results

### Primary variable—Median Error Score (MES)

**Figure 3:**
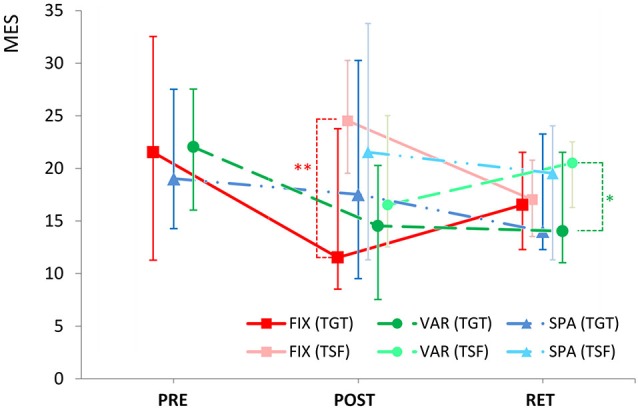
**Median error score (MES)—session- and subject-wise total number of errors across the 15 repetitions of the task; for the groups FIX (red squares with solid lines, *N* = 12), SPA (blue triangles with dotted/dashed lines, *N* = 12), and VAR (green circles with dashed lines, *N* = 12); before (PRE) and after (POST) training, and after 24 h (RET)**. Dark symbols indicate results for the target task (TGT), light symbols indicate results for the novel transfer task (TSF) for the respective groups/sessions. Error bars represent the interquartile range. ** *p* < 0.01; * *p* < 0.05 (Bonferroni-corrected pairwise Wilcoxon tests).

MES for all groups, conditions, and time points are summarized in Figure 3. Between groups, no significant performance differences could be found—neither pre-training nor POST or at RET(*p* > 0.05, Mann-Whitney). Furthermore, longitudinal within-group comparisons revealed no significant PRE-to-POST or PRE-to-RET changes due to the training session (*p* > 0.05, Wilcoxon). Immediately after training (POST), the FIX group performed with significantly more errors at the transfer (TSF) compared to the target (TGT) task (*p* < 0.006, Wilcoxon, Bonf.). Conversely, at RET, the VAR group generated significantly more errors at TSF compared to TGT (*p* < 0.048, Wilcoxon, Bonf.). No further differences were seen between the error rates in the transfer task and the target task for any group at any time point (*p* > 0.05, Wilcoxon).

### Exploratory results from secondary variables

**Figure 4:**
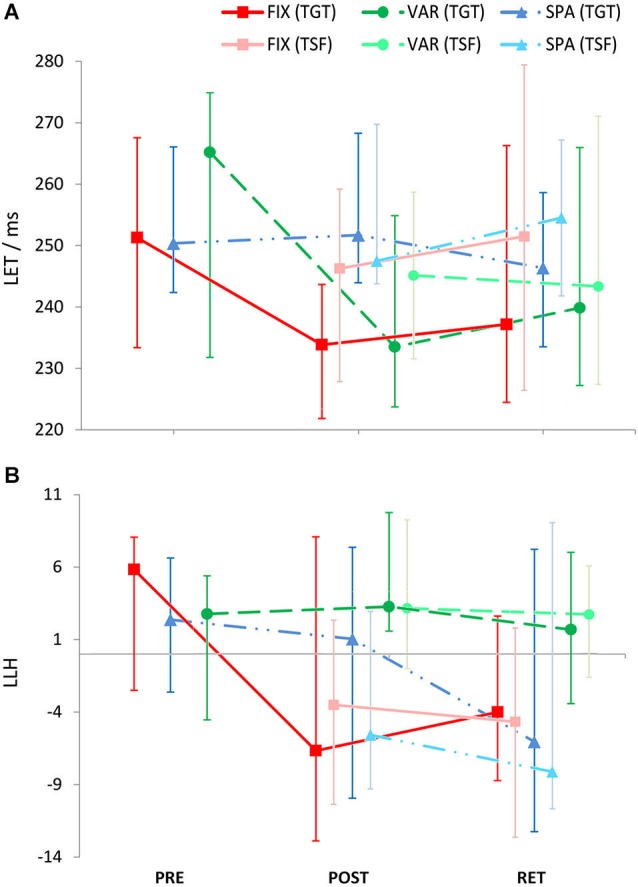
**Leap execution time (LET, panel (A)) and Leap loudness homogeneity (LLH in MIDI velocity units, panel (B)) for the groups FIX (red squares with solid lines, *N* = 12), SPA (blue triangles with dotted/dashed lines, *N* = 12), and VAR (green circles with dashed lines, *N* = 12); before (PRE) and after (POST) training, and after 24 h (RET)**. Dark symbols: target task (TGT), light symbols: transfer task (TSF). Error bars represent the interquartile range. Due to the exploratory selection of the variables LET and LLH, results are reported on a descriptive statistical level only.

**Leap Execution Time** (LET, Figure 4A) exhibited the following pattern: PRE-training, LET was similar for the VAR, FIX, and SPA groups. After training (POST), the SPA group appeared to show slower performance than both the FIX and VAR groups. FIX and VAR performed at about the same level. For comparison, the goal time for a perfectly timed leap was 187.5 ms (substantially lower than any of the time ranges in Figure 4A), re-confirming that the task was sufficiently challenging to be outside an artificial ceiling range, even after training. No performance differences between groups became emergent in contrasts involving TSF and RET conditions. The only group apparently showing longitudinal changes (learning-induced speeding up of execution time) in any of the conditions was the VAR group.

The overall pattern suggests that LET may be disregarded as a possible confound for MES, since any effect appears to be in even additive rather than in interfering direction: performances with highest MES error rates (POST performance of the SPA group on TGT and TSF; POST performance of the FIX group on TSF) displayed slowest LETs, and vice versa (POST|TGT performance of FIX and VAR groups). Hence, a slower execution of the movement does not seem to facilitate hit rate.

**Leap Loudness (LL)**. MIDI key velocity is an arbitrary scale with a value range between 0 (silence) and 127 (loudest value). Before training (PRE), all groups performed at similar velocities (not depicted; FIX: median 98.0; lower/upper quartile 79.0/107.0; SPA: 90.6; 85.5/95.0; VAR: 93.3; 82.6/98.6). After training (POST), no differences seemed to be present between groups either. During the RET session 24 h after initial acquisition, however, the VAR group apparently played louder in the TGT as well as in the TSF condition (TGT: 100.6; 89.3/104.0; TSF: 101.9; 89.0/105.0) compared to the other groups (FIX|RET|TGT: 84.9; 77.6/101.1; FIX|RET|TSF: 85.8; 76.2/98.5; SPA|RET|TGT: 87.3; 76.3/95.6; SPA|RET|TSF: 88.0; 73.1/95.2).

As with LET reported above, longitudinal changes seemed to be most pronounced in the VAR group: compared to PRE, the POST loudness was greater for both TGT (97.8; 92.0/104.9) and TSF (98.3; 89.2/105.2), and the RET loudness was greater for both TGT (100.6; 89.3/104.0) and TSF (101.9; 89.0/105.0).

**Leap Loudness Homogeneity (LLH)** data are summarized in Figure 4B. LLH is similar to the LL data since most of the differences observed in the participants’ performance seemed to be mediated by changes to the loudness in the downbeat keystroke but not the upbeat. Before training (PRE), all groups performed at similar LLH. The observation that LLH systematically tended to adopt positive values could be expected in this musical task, since it reflects that the downbeat is slightly emphasized over the upbeat. The VAR group maintained their LLH value for all conditions throughout the duration of the experiment including the second day. The other two groups, however, seemed to have dropped to negative values after the training for all remaining sessions and conditions (exception: SPA|POST|TGT 1.0; −9.9/7.4).

## Discussion

The present study aimed at the primary goal of testing whether the “VOP” hypothesis derived from Schmidt (1975) schema theory holds true for a musical learning paradigm. The task involved a challenging interval leap movement at the piano.

Participants underwent different training strategies to improve their error on the task: a group of fixed learners (FIX) received a massed practice session on the target interval. Immediately after training, this group non-significantly improved performance on the trained task but showed significantly higher errors rates at a novel transfer task compared to the trained interval; however, after a 24-h RET period, performance on the transfer task was comparable to the performance on the original task.

By contrast, the VAR group received the same amount of training but variably distributed across four different intervals (including the target). Like the FIX group, the VAR group also non-significantly improved during the training, yet the POST training error rate for the untrained transfer task was similar to the trained task. Following the RET period, target performance was still comparable to the state immediately after training, however, the error rate on the transfer task was significantly higher compared to the error rate in the target interval in the same group.

### Immediate effects of training

Hypothesis (1), namely, no immediate advantage on target performance for VAR learners over FIX learners would occur, is therefore supported in the sense that all groups performed at almost identical error rates immediately after training.

The error data at this specific time point (POST), however, may have to be taken with some caution: the lowest error scores achieved throughout the study (FIX|POST|TGT, VAR|POST|TGT, VAR|POST|TSF, VAR|RET|TSF) are all within the same narrow range (resembling an error incidence of around 0.26 per note). This might indicate the presence of some ceiling effect. Possibly, this error rate is close to rock bottom of what can be accomplished in this specific constellation (piano as secondary instrument; unfamiliar and challenging task; just one single time-limited practice session).

The theoretical underpinnings of hypothesis (1) stem from specificity-of-practice considerations (Henry, 1968; Tulving and Thompson, 1973) and from a behavioral phenomenon termed contextual interference (CI; Shea and Morgan, 1979; Shea and Zimny, 1983; Magill and Hall, 1990). According to the CI framework, in line with the VOP hypothesis, practice under increased interference conditions (like, in this case, higher variability of the training material) would lead to inferior acquisition but superior RET and transfer; and conversely, practice with low contextual interference should enhance acquisition and impair RET and transfer. A number of studies in laboratory settings confirmed this idea (e.g., Del Rey et al., 1983; Del Rey, 1989; Gabriele et al., 1989). In contrast, for ecologically valid instructional settings, several studies found support for CI only at RET and transfer (e.g., Goode and Magill, 1986; French et al., 1991; Wrisberg and Liu, 1991; Bortoli et al., 1992; Keller et al., 2006; Travlos, 2010), or reported no support for CI at acquisition specifically (French et al., 1990; Hebert et al., 1996; Brady, 1997; Jones and French, 2007).

In line with these studies, in the present data a contextual interference effect could not be observed with respect to error rates: rather than showing an initially worse performance compared to the FIX group at the end of the acquisition phase, the VAR group performed equally well right after training. It has to be noted, however, that the one (and only one) specific schedule employed for the VAR group in the present study does not allow to conclusively disentangle the two CI hypotheses. The study design aimed at the general effect of presence/absence of VOP rather than addressing the specifics of CI phenomena. The findings also imply that the specificity (SOP) hypothesis (Henry, 1968; Tulving and Thompson, 1973) is not consistent with the present data and paradigm, within the measurement sensitivity that can be obtained here given the heterogeneity of the groups.

### Retention effects

With respect to the ability to retain and consolidate the skill, hypothesis (2a) predicts a superior performance after a 24-h RET period without further training intervention in the VAR group compared to the other groups. Since the present data do not show significant changes due to the intervention in any of the groups, and, furthermore, since at RET no significant differences emerge between groups, our results are not in line with the RET hypothesis (2a). However, this does not conclusively reject the prediction and might as well simply indicate that the initial acquisition phase was not sufficiently long to generate immediate to-be-retained performance improvements in the first place.

### Transfer effects

In terms of the ability to transfer the acquired skill to a novel task within the same movement class (hypothesis 2b), the VAR learners performed the transfer task at a similar level as the original target. In contrast, the FIX learners now showed a significantly higher error rate in the transfer condition than in the trained original task.

The notion that the trained VAR group performed at the same error rate on the target interval as the FIX group is worth highlighting inasmuch as their acquired number of different content items was four times as high (compared to the FIX group). It is reasonable to assume that the three additional intervals practiced during the intervention (but not tested afterwards) would have yielded similar skill outcomes in the VAR group as the tested target interval. The FIX group, in contrast, was never exposed to these additional intervals and (if tested) might have shown similar performance as for the transfer interval that was used as the probe for transfer capabilities. Taken together, it is remarkable that after the same net amount of practice trials, the VAR group might be able to play five different intervals just as precisely as the FIX group is able to play just one of them (while probably failing at any one of the other four).

Although this appears to be supportive of hypothesis 2b, it cannot be ruled out that this strong immediate effect is due to a motor interference rather than due to an actual lack of capacity to generalize to a transfer leap within the same movement class. In other words, the failure to play the transfer task correctly may be mediated by the obstinately imposed *prime* rather than by genuine training-induced changes to the motor system. The plausibility of the “priming” interpretation receives additional support from the observation that the interference effect is washed out by the time of RET testing.

After the RET period, the VAR learners are the only group showing a significant difference between target and transfer performances. However, surprisingly the direction of the effect is opposite to what is predicted by hypothesis 2b.

It has to be noted that any effects regarding the *transfer* condition (for any data obtained in this particular paradigm) do not necessarily allow for conclusions regarding generalization of an acquired motor skill (in terms of a skill extension within the same movement class). The TSF leap (19 semitones) lies within the range of trained intervals for the VAR group (8, 12, 15, and 22 semitones, respectively). This within-range position of the novel movement was chosen in order to reflect the original barrier knock-down design by McCracken and Stelmach (1977), in which the transfer distance was in the middle of the range of training distances. However, it has been recently proposed that generalizability wears off from nearby contexts (here: distances) to distant ones along a gradient, and that therefore transfer movements are more likely to be affected when between trained distances rather than outside the practiced range (Gandolfo et al., 1996; Thoroughman and Shadmehr, 2000; Mattar and Ostry, 2007).

### Spaced practice as a control

In this study, a third intervention for another group of participants (SPA, “spaced” learners) was included in order to circumvent an interpretative ambiguity for any learning-induced performance differences on the target interval: are the putative training effects of the VAR group due to the fact that the VAR group trained additional varying material besides the target, or could they be just due to the fact that the VAR group simply spent only 25% of their practice resources on the target in question? The SPA group provided the missing link as they did the latter (practicing the target only 25% of the time, like VAR) while at the same time *not* doing the former (rather, they only practiced the target, like FIX). Since none of the within-group or between-group comparisons involving SPA yielded significant effects after correction, the following considerations are made only tentatively. In general, however, the lack of significant between-group effects indicates that neither the target nor the transfer performances in this group are different from the other two learning strategies, i.e., the present data do not show a general disadvantage of sparse practice for the task and timeframe under investigation.

An interpretation of the SPA results in the POST session is difficult: the overall level of (a) cognitive load and interference; (b) distraction; and (c) fatigue effects are probably different between the SPA group and the VAR/FIX groups. Interference effects between a motor task and a secondary task may occur if one or both of the tasks require sufficient attention and the two tasks share limited resources (Schmidt, 1988; Frensch et al., 1998). Imposition of a cognitive load has been demonstrated to interfere with simple visuomotor adaptation (Redding et al., 1992; Taylor and Thoroughman, 2007). Moreover, the learning process takes place trial-by-trial, in the sense that an error in one trial informs the subsequent movement (Thoroughman and Shadmehr, 2000; Scheidt et al., 2001; Thoroughman and Taylor, 2005; Fine and Thoroughman, 2006). Hence, the long stretches of intermittent distraction in the SPA group through the additional reading task may have provided additional cognitive load and washed out valuable feedback information. On top of this, the SPA group was subjected to an attention divide (which has been shown to be detrimental to transfer challenges specifically, Bédard and Song, 2013; Song and Bédard, 2013). An additive effect was not observed in the data.

The final outcome (RET) of the SPA intervention is similar to VAR with regard to error rate (TGT and TSF) and, on descriptive level, appears slightly slower than any of the other groups with regard to execution times, and similar to FIX with regard to loudness. Therefore, it might be the case that spaced learning combines the “worst of both worlds” with respect to variable and massed-fixed training strategies, respectively.

### Observations on secondary variables

The investigated secondary variables are not intended to represent a report of scientifically robust results. This study aims at empirically extending the idea of VOP to musical practice. Therefore, while being able to objectify hypotheses concerning the primary variable, in the current setting the quest for ecologically meaningful secondary variables is exploratory. In the following, the main observations on secondary variables are summarized and putatively formulated as predictions that may be addressed in future research:

1. Massed practice (regardless of whether it introduces variability or not) leads to an overall increase of motor execution speed immediately after practice, and after a RET period.
2. Variable practice schedules increases the execution speed to a greater extent than do constant or fixed schedules (also consistent with previous observations in a related paradigm, Stambaugh, 2011).
3. Variable practice will result in an increase of loudness (mechanical force/velocity) applied to the instrument and may thus reflect a build-up of confidence regarding the acquired skill, both in the practiced condition as well as in novel challenges from the same movement class.
4. Conversely, a fixed training intervention (regardless of massed or sparse schedule) leads to a substantial (immediate and retained) decrease of applied forces, for both the trained task and similar novel tasks. In the case of the piano interval leap, this might be interpreted (a) as a detrimental effect on self-evaluated confidence at the skill; or (b)—because of the correlation of key velocity and terminal speed of the moving hand –, as a positive indicator of heightened precision motor control compared to variable learners (i.e., slowing down in the crucial final phase of a motion while simultaneously preserving the overall average movement speed).

### Limitations and future extensions of the current design

#### Subject selection

As is reflected in the error bars (quartiles) of Figures 3, 4, the overall level of performance was notably heterogeneous between subjects: for example, a descriptive statistic on MES across all three participant groups revealed that in the initial, untrained state (PRE), the median MES was at 21 errors, with the weakest performer at 57 errors, and the strongest performer at only 2 errors. In other words, the distribution of the subject sample spans a range of 55 errors. Therefore, any inferential statistical evaluation was bound to objectify possibly fewer effects than what might be expected from a more homogeneous pool of subjects. Moreover, factoring out inter-individual baseline differences by looking at the learning-induced individual *changes* only would not narrow the distribution range either: the POST-to-PRE score difference was distributed between a maximum of +41 additional errors after training compared to before training, and a minimum of −23 errors in the best learner. This additionally indicates that participants might as well be differentially susceptible to specific types of training strategies like fixed vs. variable schedules. Table 1 indicates a high degree of heterogeneity of e.g., the cumulative hours of lifetime practice at the piano (being the secondary instrument). It has been demonstrated that certain experience-related characteristics of individuals may well have an impact on how their motor systems respond to variability of motor learning schedules (Wrisberg and Mead, 1983; Ollis et al., 2005).

#### Duration of intervention

The design of this study involved the possibly rather limited impact of a single and short training session. In the motor learning literature, interventions typically range up to several weeks. Studies with a more ecologically valid schedule—although difficult to control—may involve multi-session training with long-term RET testing (Giuffrida et al., 2002; Bangert and Altenmüller, 2003; Savion-Lemieux and Penhune, 2005).

#### Schedule architectures

The present study focuses on the main effect of impact of the degree of variability introduced into the training of a motor program, which is achieved by varying the number of different items to be rehearsed. However, CI approaches early-on have introduced schedule patterning into their paradigms (for an early review, see Magill and Hall, 1990). In short, the idea is, while keeping the number of different items the same across groups, to vary the *relative order* of items within the schedule in order to achieve different degrees of contextual interference (e.g., random, serial, blocked). Gradual increasing CI within-schedule has been shown to affect the outcome positively (Feghhi and Valizde, 2011; Saemi et al., 2012). Surprisingly, this exhaustive body of research has yet only rarely been systematically extended to music (Rose, 2006; Stambaugh and Demorest, 2010; Stambaugh, 2011) despite the apparent implications such findings may bear for music education. The present comparison of FIX and VAR interventions tackles the effects of VOP only (i.e., presence vs. absence of variations in the practiced material) and does not address effects of scheduling (variations of serial ordering or blocking within otherwise identical material). The VAR intervention involved only one type of schedule (blocked-random), which was chosen after several piloting runs of the paradigm as being the one subjects felt most comfortable with. The third group (SPA), although suggestive of being a schedule variation of FIX, merely serves as an intermediate control. This group is in close correspondence to the identically designed control group first introduced in McCracken and Stelmach (1977) landmark research on VOP.

#### Knowledge of performance and knowledge of results

The present study has not addressed the specific contributions of Knowledge of Performance (KP) or Knowledge of Results to the observed effects. While KP refers to the instant sensory feedback inherent to a motor action and practice situation, Knowledge of Results (KR) addresses offline forms of abstract or quantified information provided after a practice trial, like, in instructional settings, the verbal feedback given by an educator. The current design implies a characteristic feature of music instruments in that they provide intrinsic KP feedback for each single trial, thus often rendering KR redundant (Schmidt and Wrisberg, 2004). Explicit KR was not offered to the participants during the learning.

A large body of literature provides compelling evidence that systematically providing KR during the training enhances the effects of motor learning (McNevin et al., 1994; Guadagnoli et al., 1996; Shea and Wulf, 1999; Fredenburg et al., 2001; Guadagnoli and Kohl, 2001; van Vliet and Wulf, 2006). Specifically, the effects of variations of KR frequency (Salmoni et al., 1984), feedback manipulations (Ishikura, 2005; Hatfield et al., 2010), or augmentations (Wu et al., 2011) can be studied utilizing the technical versatility of digital musical instruments.

#### Aesthetic considerations

Our analysis focused on quantifiable technical aspects of playing accuracy. Subsequent studies should also strive to find ways to objectify the assessment of esthetic parameters like ratings of quality/musicality of the performance (cf. Stambaugh and Demorest, 2010; Stambaugh, 2011).

### Overall feasibility of the Leap Task as a probe for musical motor skill learning

This is the first study of its kind utilizing a relatively simple goal-directed movement in a musical context. Usually, task material exploited in motor learning studies of music tend to be significantly more elaborated (melodic lines or excerpts of instrumental literature, often bimanual in the case of pianists) in order to reflect actual real-world demands of music motor behavior. Previous findings suggest that the effects of VOP may depend on the qualitative properties of the task in training and transfer (Healy et al., 2006; Boutin and Blandin, 2010). In addition to the nature of the task, several empirical accounts have indicated that the degree of complexity of a task is of crucial relevance (see Wulf and Shea, 2002). As complexity increases, learners seem to benefit more from the opportunity to repeat and refine their responses on successive trials. Consequently, CI effects tend to be reduced or eliminated with tasks that are more complex. Since music provides an ideal material to allow for systematic variations of complexity level, future research may address the effects of variable vs. fixed practice in more complex musical tasks.

As for the overall methodological feasibility of using a single-shot goal-directed movement as a means of quantifying learning-dependent performance in a musical context, the present data provide evidence of the reliability and replicability of the measurement. The wide distributions of group data are a mere result of group heterogeneity rather than inherent to the measurement approach: for error rates, the average intra-cluster correlation coefficient was determined to be *ρ* = 0.748 across the 15 repetitions of the leap within condition/within subject, indicating a high degree of robustness of the approach.

## Conclusion

This study was performed to extend the research on variability-of-practice phenomena to the field of musical instrumental practice. In the specific task under consideration, variable practice compared to fixed practice, while devoting only one quarter of the practice time to the target interval, showed training results comparable to the outcome of two non-variable training interventions, in target, transfer and RET conditions. Contrary to the prediction hypothesized by VOP theory, however, the participant group employing variable practice (unlike the other groups) produced significantly more errors in the transfer compared to the target task at RET testing. As a limitation, none of the participant groups measurably improved on the primary variable (error rate) after only one training session. Thus, the results provide only partial support for the validity of the VOP hypothesis for the given setting. In order to reach conclusive results, additional research is needed that addresses the recommendations derived from the current data, namely (1) a more homogeneous participant sample; and (2) a more extended multi-session training intervention.

From a more general perspective, in many real-world musical challenges certain benefits of variability (if the task allows for its use) cannot be overstated: firstly, greater diversity of the tasks may allow learners to extract the most relevant, task-invariant information. Especially in the initial stages of becoming acquainted with a new instrument or a new technique, this might facilitate faster training progress in the mid- and long-term. Using variable rather than rigid movement material has been shown to recruit additional brain areas like prefrontal cortex (Kantak et al., 2011), which might give a hint that there are physiological differences in the neural underpinnings of the different training strategies. Secondly, VOP does not only have consequences for the brain but also for the body: because variable learners are frequently changing tasks, they potentially reduce the risk of physical problems, like overuse syndromes and other medical issues induced by highly repetitive motor activity. Finally, a crucial advantage of VOP may lie in its effects on motivation. Variability potentially counteracts feelings of boredom, heightens the level of engagement (Simon and Bjork, 2001), and provides learners with a larger “playground” to populate with the full parameter range of (in case of music) expressive abilities to draw from.

## Author and contributors

Each of the authors listed has made substantial contributions to the conception or design of the work (Marc Bangert, Anna Wiedemann, Hans-Christian Jabusch), or the acquisition (Marc Bangert, Anna Wiedemann), analysis (Marc Bangert, Hans-Christian Jabusch), and interpretation (Marc Bangert, Hans-Christian Jabusch) of data for the work. All authors were involved in drafting the work or revising it critically for important intellectual content, in the final approval of the version to be published, and consented to be accountable for all aspects of the work in ensuring that questions related to the accuracy or integrity of any part of the work are appropriately investigated and resolved.

## Conflict of interest statement

This research was conducted in the absence of any commercial or financial relationships that could be construed as a potential conflict of interest.

## Footnotes

^1^www.neurobs.com
